# Extract from Prof. Eve’s Valedictory

**Published:** 1855-09

**Authors:** 


### EDITORIAL DEPARTMENT.

### EXTRACT FROM PROF. EVE'S VALEDICTORY.

Prof. Paul F. Eve, of Nashville, has delivered one of his usual spirited and eminently professional Valedictories to the late large class of the College in which he teaches. We quote the following in reference to the meddling of the clergy with medical science, with the simple remark, that a member of the Philadelphia County Medical Society was lately near being expelled, for making one of these gentry pay him a moderate fee for the performance of an important and successful surgical operation!!

"Of all persons, one would suppose the clergy ought most to co-operate with the medical profession. There are certainly many good reasons why the two callings should unite harmoniously, and while mutually assisting each other, each in its own sphere of duties, never to come in collision. Reid said, years ago, the medical cannot be seperated from the moral science, without reciprocal and essential mutilation. And who, let me ask, are provoking this separation? I yield to no man in love and veneration for, or in honoring and sustaining, the humble, conscientious and devoted minister of God; but as for the hypocritical professor, who leaves his own calling to interfere with ours, and uses religion to cloak his designs, I consider him the worst enemy to both. One would suppose the clerical profession had enough of its own polemics to settle, without volunteering to determine the true science of others. If more faithfully and harmoniously engaged about their own all-important, holy calling, the apprehension entertained by many might be relieved, that some of the controvertists so fall out with each other concerning the true road to heaven, as never to reach their journey's end."

The above is taken from the *Philadelphia Medical and Surgical Journal*, to which we unite our testimony of the excellency of Prof. Eve's valedictory. He invites the attention of the profession to a subject of no little importance to its best interests.

There is certainly a very close relation existing between the medical and the moral sciences. They are so closely allied as to be regarded, by the intelligent and informed, as handmaids to each other in the great office of good to society. There is a law of society—a supreme and a universal law, suitable for civilized and enlightened man under all circumstances, and in all ages. It was the gift of inspiration, and written through the instrumentality of the ancient "law-giver." This rule or law, the inimitable decade was not suggested by his knowledge of mankind or its moral necessities. He was therefore prompted by inspiration and made the medium of revealing to man this divine rule or code for his civil, social, and domestic government while in relation to his fellow-men.

For the same reasons we are allowed to infer that he was also inspired to proclaim physiological truths, which are now taught by all sound orthodox medical men. The declaration that "the life of the flesh is the blood thereof," was as much the result of inspiration as the divine decade itself, for if he knew not the moral wants of society, in all ages to come, he knew less of the physical structure or their functional qualities, because dissections had not been practiced. It was not hypothesis, it was wisdom taught him by inspiration, and is as much a truth as the decade is truth. If it be heterodox to deny the truthfulness or divinity of the decade, it is also heterodox to deny the truth or the inspiration which promted the avowal of the above physiological truth. They were uttered about the same time and, although not engraven on stone, were nevertheless recorded as other divine truths are found recorded, preserved and mingling in the context. Admit the fact that medicine has undergone many mutations and changes, and that it lost itself as it were, in witchery and blasphemous incantations.—The sacred decade, too, with literature and science generally, were also all hidden in the gloom of the dark ages, but came forth through well known reviving and vivifying influences. With the revival of letters, science came forth also, and medical science among the others. It continued onward, improving from time to time, and its advancement was coeval with the progress of civilization. Christianity and medical science progressed together, side by side, and thus coupled they appeared as kindred systems. Physiology progressed and improved in proportion to the opportunities of investigation and the progress of research, and in its onward progress the truth of the inspired writer was now clearly revealed. The regular system of medicine was that which progressed forward, kept pace with progress, confirmed original truths, and rejected ancient errors after showing their inappitency and falsity.

We claim then, to be, and no one will exhibit his want of liberality and intelligence by denying our unqualified right to it, the ancient system, in existence for thousands of years, which system has been sanctified by time as well as by its humane and benevolent offices. If we teach the truths which were once proclaimed by inspiration, and if the history of our system is plainly traceable back to the early records of the world, then it would follow that our's is an institution of divine appointment for the relief, as far as human agency can go, of our afflicted fellow man. If this be true, then it would be *heterodox* to oppose it and would be *infidelism* to avow any other doctrine.

But infidelism in religion is a reproach to those who advocate so dangerous a doctrine, say those whose duty it is to teach divine truth and point the sinner to repentance. The bible is the rule of faith, they persist, and it is heterodoxy to disbelieve its teachings. How, and with what fervor the various false systems and *isms* are denounced, with those who follow after them? How eloquently is truth portrayed, and how black is error made to be by the graphic description of the learned divine? All well, and all right, say we.

But let us inquire how many of this class of christian believers are there who are *infidels* in medicine? How many of them are believers in the various forms of empiricisms, embracing the different *isms* and *schisms* now turning the brains of the people with regard to medicine? For every *ism* in religion we can find one in medicine, for every *fanatic* system of theology we can find some *unbelief* in medical doctrine.

The infidelity is almost the same, the one betraying as much disregard for reason and truth as the other. But woe to him who believes in Mormorism, with its insulting and grossly immoral polygamous practices, and how much are *they* pitied and deplored who are led off by Millerism and its quick-coming consummation of all things!

It is as much to be lamented as it is true that many of those who warn their followers most zealously against these dangerous systems, are the advocates and propagandists of the sheerest humbugs, the basest cheats, and the most untenable and erroneous doctrines ever advocated by rational and intelligent men. Their powerful control over the minds of their followers is very often used in favor of their darling system, whether Eclectic, Thompsonian, Homoeapathy, Hydropathy, the *ism* of recent birth, *fapo-o-pathy*, or some other of the "seven head and ten horned" infidel systems, scarcely furnishing claims to support, should the world be again enveloped in the oblivious condition of another Cimmerian night, when fanaticism and mental darkness shall shroud the mind and obscure the intellect.

We declare it to be our deliberate conviction that, if it were not for the vitalizing influences imparted to these several systems by some of those to whom the world looks with confidence for spiritual advice, that empiricism would present but a meagre spectre and if found at all it would be in the haunts of mental darkness, the depths of ignorance, and the home of blind superstition.

The world is strongly inclined to doubt the wisdom and judgement of those found in the chase after every will-o-the-wisp, which shows its phosphorescent colors to mislead and betray. There is a species of fickleness manifest in such persons, for the last which springs up is brightest and best, a kind of *sine qua non*, the very paragon of super-excellence. All preceding delusions are abandoned and the pursuit of the new is characterized by hot zeal and to be changed only upon the appearance of some new delusive light. Thus they go, led blindly, but yet led on, and on, from time to time, and such their mental appetite and such their perverted judgments that old truths become tame, tasteless, and insipid.—They want the spice of novelty, with the emotions which it always inspires to awaken an interest as much as a spoiled child indulged in sweetmeats, needs highly seasoned food to make an impression upon the sense of taste. With such there is a restless discontentment, they look for perfection in every thing with credulity enough to believe every new delusion is a specimen of perfection.

The firm believers in the christian faith, the true members of the medical profession well know that in the depths of our science there has been furnished more confirmatory testimony in favor of its truth, than in all the other departments of science together.— The sound medical teacher will furnish stronger illustrations in support of the truths of the christian faith than found in any other department of science. They therefore contribute largely to the force arrayed against materialism and infidelity, and yet notwithstanding there are certain clergymen to be found who are loud in their condemnation of the regular and active in foisting into public notice and favor their favorite medical dogma.

Men essentially wrong in one particular, are prone to err in others particularly, where there is a tendency in the same direction. It is always better to leave each department to itself, to its own management, and in this particular medical men are remarkable. It is seldom that a learned medical man finds fault with the duties of the clergy or with the doctrines he teaches, so that they are orthodox, and he never meddles himself with them in the discharge of their duties. We ask for the same exemption and if we are denied it by community generally, we ask it especially from the learned divines. We accede to them a better understanding of the scriptures and the moral wants of community, and therefore leave, uninterrupted, the matter with them to direct. But while we yield to them in this particular, we claim that we too have burned the midnight lamp, closeted ourselves in the dissecting room with its mephitic air, have clung to our profession with a devotion amounting to an idolatrous worship, and claim the right we cheerfully accede to them—the right to know more and better of medicine than they. An imposter of a single month's manufacture is galvanized by these meddlers by their clerical influence, into a *beau ideal* of perfection. How absurd and illiberal such a procedure.

We do not wonder that the uninformed are misled and misguided by clap-traps in medicine because the artful management of the authors of them, is well designed to deceive the unwary. But when we see men claiming to possess a liberal education and large mental endowments, made the willing victims of chicanery, we feel that we have loud cause for complaint and very good reason for doubting their sincerity in any thing. Those engaged all the while in an analysis of the conduct of men and quick to detect error and the secret motives which prompt its execution, should certainly be able to detect, at first sight, the hollow artifices of the disciples of the several side-systems. We complain of such the more loudly, because a large number, governed by considerations of good to their fellow men, are zealously active in promoting the advancement and cultivation of medical science. For such magnanimous and pious men, these remarks are by no means meant or intended. We observe that these divines always select men of the highest capabilities and of the most liberal culture in medicine, in such they repose all the confidence, which their consciousness of human imperfection will allow them, in their wisdom and management. True medical men always receive from such considerations of respect and regard—dictated by an appreciation of their usefulness and efficiency in society.

No, we direct our remarks to that class we have plainly indicated and from whose intermeddling in the peaceful discharge of our duties, we desire to be relieved. This desire is dictated by considerations of humanity and not by selfish considerations. It is not from pecuniary considerations, because in that regard we are rather benefited by the existence of empiricism. Our motives are of a more elevated character and find their origin in a desire for the good of society. Nor is the dignity of the profession lowered, on the contrary, it is elevated by contrast with the low grade which these medical mountebank systems occupy. Virtue is dignified in proportion as vice is degrading. The one is admired for its purity and holiness in proportion as the other is hated and contemned.—It is therefore no selfish consideration that prompts these recorded thoughts, but for the welfare of the human family, whose health and life are jeoparded every day by quackery, that we appeal. The very life blood of these empirics is gleaned from men who are prominent in society and who would show their consistency just as well if they would aid and counsel the plunderer upon the highway or the pirate upon the high seas. Essentially it is the same, with this difference in the enormity of the offences,—the one is done openly and boldly, the other is done through deception and hypocrisy. In some particulars it is even worse, for the robber would avoid the destruction of innocent children and delicate females, but the empiric makes no discrimination. How terrible must be their retribution for the sin of experimenting upon children, with nostrums without the ability to protect themselves. This is done wilfully too often because the disciples of some of these isms must know they are practising hypocrisy and deception. Therefore no honest man can be found practicing them, because they *cannot* believe one iota themselves in the tenets which they propose to carry out into practice.

Conscientious and intelligent clergymen should bring the subject before their ecclesiastical assemblies for action, the error exposed, and the practice condemned. This course would do much to cure the evil of which we complain, and also relieve the body from an approbrium which pertains to it in consequence of the conduct of some of its members. We have spoken plainly and will continue to do so, but in a spirit of kindness and far from all ill-feeling. We are the friends of the clergy and do our share for their support while we listen with attention to their teaching. But a decent respect for ourselves and a humane regard for our fellow men, demand of us that we defend humanity against the practices of the empiric and ourselves against antagonistic influences.

Medical men do not generally charge clergymen for advice or services rendered to themselves or families and are pleased to contribute, as far as possible, to their well being and safety, but as a learned body of men, we claim an equal respect, confidence, and favor at their hands, instead of a warfare against a system acknowledged for centuries to be efficient for good in their arduous and responsible vocation.

